# Is Retail Extra Virgin Olive Oil Truly Extra Virgin? Consumer Perceptions Versus Analytical Quality Across Price Ranges

**DOI:** 10.1002/fsn3.70471

**Published:** 2025-06-22

**Authors:** DeSantis Diana, Ferri Serena, Barelli Benedetta, Cagnazzi Lavinia, Modesti Margherita

**Affiliations:** ^1^ Department for Innovation in Biological, Agro‐Food and Forest Systems (DIBAF) University of Tuscia Viterbo Italy

**Keywords:** declared quality, expected quality, labeling transparency, quality assessment, regulatory standards, sensory defects

## Abstract

In recent decades, extra virgin olive oil demand has grown globally. The increasing popularity has led to a highly diversified commercial offer: supermarkets and discounters present a wide variety of brands and price ranges. However, price does not always correlate with quality, and label information can often mislead consumers. Understanding consumer preferences, knowledge, and drivers of perceived quality is crucial for maintaining trust in the extra virgin olive oil sector. This study investigates the alignment between consumers' expectations based on label information and the actual quality of extra virgin olive oils through a commercial and chemical analytical screening. The research objectives included a consumer survey to identify purchasing drivers, consumption habits, and knowledge of quality standards; a commercial survey on the extra virgin olive oil available in different stores, assessing brand, price variability, and transparency of information; and chemical and sensory analyses on selected extra virgin olive oils of different price ranges to evaluate whether the declared quality reflects the actual quality. The results showed significant inconsistencies in quality: although all oils were labeled as extra virgin olive oil, many medium‐ and low‐priced samples presented sensory defects that EU regulations prohibit for extra virgin olive oil. Notably, oils with more detailed and transparent labeling, such as harvest year, cultivar, and extraction method, tended to perform better in chemical and sensory analyses, regardless of price. This suggests that consumers may better assess quality by scrutinizing label information rather than relying solely on price, highlighting the practical importance of comprehensive labeling.

## Graphical abstract

This study evaluates whether retail extra virgin olive oils meet quality expectations based on consumer perceptions, label information, and price. Combining consumer surveys with chemical and sensory analyses of extra vergin olive oils, we reveal significant discrepancies between declared and actual quality, particularly in lower‐priced oils. Our findings emphasize the need for improved labeling transparency and consumer education to enhance trust and informed purchasing decisions.

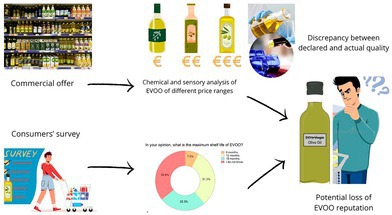

## 1 Introduction

Extra virgin olive oil (EVOO) is a cornerstone of Mediterranean cuisine and culture, widely appreciated for its sensory qualities and health benefits. Its consumption has expanded globally in recent decades, driven by growing recognition of its unique flavor profile and nutritional value (Covas et al. 2009; Rébufa et al. 2021). Italy plays a key role in the EVOO market, ranking second in global production (after Spain), with exports of approximately 300,000 tons per year (International Olive Oil Council 2024). At the same time, Italy is the world's leading EVOO consumer, with a per capita consumption of 12 kg per year, requiring the country to import over 500,000 tons annually to satisfy domestic demand (FAO 2022). Italian consumers are generally recognized as being extremely informed about EVOO quality and composition. This is due to their cultural connection to olive oil, proximity to production areas, and the prevalence of quality certification schemes such as the European Union's Protected Designation of Origin (PDO) and Protected Geographical Indication (PGI) (Latino et al. 2022). Educational campaigns, olive oil festivals, and the cultural prominence of the Mediterranean diet further reinforce consumer awareness and interest in EVOO (Cacchiarelli et al. 2018).

Despite this, the rising global demand has also intensified the commercial offer, which is highly diversified and confusing to consumers. Supermarkets and discount retailers offer a broad spectrum of EVOO products of different price ranges. As such, consumers, faced with many choices, often rely on price and label information to guide their purchases. Unfortunately, price is not always a reliable indicator of quality. Moreover, current labeling practices frequently lack essential information, such as the harvest year, making it difficult for consumers to assess product freshness and quality accurately. While regulations permit the omission of certain details—such as the harvest year unless all olives are from the same crop year—this regulatory gap can mislead consumers into equating “best‐before” dates with product freshness (European Commission 2022).

Regulatory standards for EVOO classification require stringent chemical and sensory parameters, including low levels of free acidity, minimal peroxide values, and the absence of sensory defects (International Olive Council (IOC) 2019). However, there is growing concern that these standards are inconsistently applied and enforced, particularly in large‐scale retail distribution (LSD) channels. Studies have demonstrated discrepancies between what EVOO labels claim and the actual quality of the product, leading to potential consumer deception, loss of trust, and damage to the sector's credibility (Valli et al. 2016).

Recent advances in traceability and authentication technologies (Calò et al. 2022) are increasingly being used to ensure the transparency and origin of olive oil products (Calò et al. 2022). In parallel, novel instrumental approaches like the electronic nose and electronic tongue are gaining recognition for their ability to support or complement traditional sensory panels in quality assessment (Buratti et al. 2018).

Given EVOO crucial role in the global agrifood sector, and considering the positive consumption trends, it is critical to investigate whether consumer expectations based on label information and price align with the actual quality of EVOO available on the retail market (Sgroi et al. 2024). This study addresses two main issues: (i) the transparency and accuracy of EVOO labeling in large‐scale retail settings and (ii) the alignment (or misalignment) between consumer expectations—shaped by label information, price, and origin—and the actual chemical and sensory quality of EVOO. To achieve this, the research was structured around three key steps. A consumers' survey to explore Italian consumers' purchasing habits, drivers of choice, attention to label information, and knowledge about EVOO quality standards; A census of EVOO products available in two hypermarkets and three discount stores in Central Italy (Lazio region), focusing on brand diversity, price, origin, and label information and, lastly, an analytical (acidity, peroxides value, polyphenols, volatile profile and electronic nose (E‐nose) measurement) and sensory evaluation on selected EVOO samples from different price ranges to verify whether the declared quality aligns with EU regulation and actual quality.

## 2 Materials and Methods

### 2.1 Consumers Survey

Data were collected in September–October 2023 by way of a nationwide online survey with Italian consumers. The survey was created and administered by researchers of DIBAF, University of Tuscia (Viterbo, Lazio Region, Italy). Based on the purpose of the research study, the survey has been carried out only among Italian olive oil consumers and therefore the respondents were first asked about their EVOO consumption. Only those respondents who indicated they consumes EVOO (even occasionally) were allowed to proceed with the questionnaire. The questionnaire included an initial section consisting of closed ended questions regarding consumers sociodemographic profile. The second section focuses on EVOO purchasing habits and the last section on the purchasing motivations drivers and EVOO Knowledge. Appendix S1 presents all the questions, together with their scales. All consumers voluntarily agreed to participate in the study and were assured that their answers would be kept confidential and completely anonymous. Although a total of 275 questionnaires were received, the final number of valid questionnaires used was 266, following the exclusion of nine interviews mainly due to incomplete answers.

### 2.2 Commercial Offer Analysis

In the second phase, a commercial survey on EVOO offerings from five retail outlets in Viterbo (Lazio Region, Italy) has been carried out. Lazio, a region renowned for its olive oil production, represents an area with a long‐standing tradition of EVOO production and consumption, playing an important role in the national olive oil production landscape. The survey included the commercial offer of two hypermarkets and three discount stores. Data were systematically collected in a purpose‐built Excel sheet that recorded the number and diversity of available brands, country of origin, presence of DOP or Protected IGP certifications, price per liter, olive harvest year, packaging type, and details on extraction methods as indicated on product labels.

### 2.3 Chemical Analysis

The last phase included the chemical and sensory analysis of selected EVOO to verify the correspondence between EVOO labels information and actual quality. Eight EVOO samples from commercial sources were selected from the cataloged offerings, and three artisanal oils were obtained directly from local mills. The 8 commercial samples were chosen to cover a range of price tiers (low: under €7 per liter, medium: under €14 per liter, and high: between €15 and €25 per liter) and included both supermarket and discount store products, aiming to represent the diversity of the retail offering. The 3 artisanal oils were directly sourced from regional mills to serve as a quality benchmark and to explore whether shorter supply chains and more traditional practices influence quality. Low, medium, and high tiers were designated by LP, MP, and HP codes, respectively (Table 1). All selected samples were from the 2022/2023 harvest and commercially classified as EVOO. Free fatty acid (FFA) expressed in % of oleic acid, and peroxide value (PV) (meq O_2_ × kg^−1^) were carried out according to the official analytical methods (International Olive Council T.20 Doc. No 34/Rev. 1, 2017; International Olive Council T.20 Doc. No 35/Rev. 1, 2017). Total phenol content (TPC) was performed as described by Waterhouse (Waterhouse 2002), with minor modifications (De Santis et al. 2024). Measurements were performed using a spectrophotometer (Lambda 25 UV–Vis, Agilent, Santa Clara, USA) at 760 nm. TPC was expressed as mg gallic acid equivalent (GAE) per kg of oil sample, based on a calibration curve obtained with known concentrations of gallic acid (De Santis et al. 2024).

**TABLE 1:** EVOO selected for chemical and sensory investigations with their respective price per liter, location of purchase (retailer), price tier, certification, extraction and filtration, and variety.

	Price (€ /L)	Retailer	Price tier	Harvest year	DPO/PGI	Extraction/filtration	Origin
LP1	5.18	Hypermarket	Low	2022/2023	None	Filtered	Blend UE
LP2	5.99	Hypermarket	Low	2022/2023	None	Filtered	Blend UE
LP3	6.65	Discount	Low	2022/2023	None	Filtered	Blend UE
MP1	10.00	Hypermarket	Medium	2022/2023	None	Filtered	Blend UE
MP2	11.50	Hypermarket	Medium	2022/2023	None	Filtered	Blend Italy
MP3	10.99	Hypermarket	Medium	2022/2023	None	Not Filtered/cold extraction	Blend Italy
MP4	9.98	Discount	Medium	2022/2023	IGP	Filtered/cold extraction	Blend Italy
HP1	22.50	Hypermarket	High	2022/2023	None	Filtered/cold extraction	Blend Leccino, Frantoio, Moraiolo
HP2	25.00	Local mill	High	2022/2023	None	Filtered/cold extraction	Monocultivar Leccino
HP3	24.00	Local mill	High	2022/2023	None	Filtered/cold extraction	Monocultivar Caninese
HP4	24.00	Local mill	High	2022/2023	None	Filtered/cold extraction	Monocultivar Caninese

### 2.4 Aromatic Profile Evaluation

Head space gas chromatography–mass spectrometry (HS GC–MS) was used for in‐depth volatile organic compounds (VOCs) analysis. Analyses were conducted using a PerkinElmer Clarus SQ 8 GC–MS system equipped with a PerkinElmer Turbomatrix HS‐40 headspace sampler. Each sample (5 mL) was incubated in 20 mL headspace vials, sealed with crimped aluminum caps featuring PTFE‐lined silicone septa. Detailed specifications of HS GC–MS conditions are reported in Table S1 Volatile identification was achieved using the Amdis (2003 Tobias Kind) and National Institute of Standards and Technology (NIST) Mass Spectral Database (NIST 98, Version 2.0, USA). Only compounds with an 80% match or more were selected. Selected peaks were then quantified using TurboMass software (TurboMass R, Version 5.4.2 PerkinElmer Inc., USA, 2008), by integration of the peak areas. The area of each peak was then normalized to the sum of the areas (Modesti et al. 2024).

Additionally, the volatile composition of the selected EVOOs was also analyzed through an E‐nose, assembled at the University of Rome Tor Vergata. It comprises an array of 12 quartz microbalances (QMBs), sensitive to mass changes. The QMB array was functionalized with specific porphyrin‐based metal complexes—using metals such as Mg Co, Cu, Zn, Fe, Mn, and Sn—as well as free‐base porphyrins, enabling selective interaction with VOCs. When volatile compounds from the oil samples interact with the QMB sensors, small mass changes on the sensor's quartz surface cause a proportional frequency shift in the oscillator circuit output, with each QMB sensor achieving nanogram‐level mass resolution. Each sensor was individually connected to an oscillator circuit, with a temperature‐compensated crystal as a reference, achieving frequency resolution up to 0.1 Hz (Capuano et al. 2023). For each analysis, 5 mL of the EVOO sample was placed in a 20 mL vial and incubated at 30°C for 20 min. Headspace volatiles were then extracted for 90 s using filtered air and directed into the E‐nose chamber. Post‐analysis, a 300 s pure air stream reset the baseline (Muñoz‐Castells et al. 2025). Sensor responses were recorded as frequency shifts, generating unique “fingerprint” patterns that characterize the volatile profile of each oil sample.

### 2.5 Sensory Evaluation

According to Commission Regulation (EU) 1604/2019 (Council International Olive Oil 2018), an olive oil's sensory characteristics are considered compliant with its declared quality category if classification is confirmed by an official sensory panel. The Council International Olive Oil official method was used for sensory analysis using a standard profile sheet (Council International Olive Oil 2018) (Figure S1). The intensity of the different attributes was scored on a 10 cm intensity scale. The standard profile sheet contains three positive attributes (fruity, bitter, and pungent) and five main negative attributes (fusty/muddy sediment, musty/humid/earthy, winey/vinegary acid/sour, frostbitten olives, rancid and other negative attributes). The panel consisted of 10 judges trained and certified for organoleptic evaluation of EVOO (Council International Olive Oil 2021). The tests were conducted in a tasting room, on a 15 mL of oil, in standard blue tasting glass (Council international Olive Oil 2020), covered with a watch glass, and conditioned in a heater electric (Panel‐Test Mod.145, Ettore Pasquali srl, Italy) at 28°C ± 2°C temperature.

### 2.6 Data Processing

All the data analysis were performed in triplicate. Chemical data were subjected to the Bartlett test and Shapiro–Wilk test to verify normality and homogeneity of the variances. Once these prerequisites were verified, one‐way ANOVA and Tukey's test at *p* ≤ 0.05 was performed by using GraphPad Prism (2024 GraphPad Software, Boston, MA, USA). GC–MS data were autoscaled and used for a principal component analysis (PCA) with Venetian blind as validation method (with blind thickness = 1). E‐nose numerical data were autoscaled and then analyzed with partial least square discriminant analysis (PLS‐DA). To ensure robust validation of the model and avoid overfitting due to replicates, we implemented a leave‐one‐group‐out cross‐validation (LOGO‐CV) strategy. In this approach, each original sample (e.g., HP1, LP2, etc.), which was measured in duplicate, was treated as a unique group. During cross‐validation, all replicates from a given sample were excluded simultaneously from the training set and used solely for testing. This strategy prevents data leakage between training and testing phases, which can otherwise occur when duplicate observations of the same sample are split across folds. Such leakage would inflate model accuracy due to the artificial similarity between training and test data, leading to over‐optimistic results. Multivariate analyses were performed by using Matlab R2013a (MathWorks, Natick, MA, USA) and PLS Toolbox (Eigenvector Research Inc., Manson, WA, USA). Sensory data were analyzed using the official method (Council international Olive Oil 2024), which includes calculating the median (Me) as the 50th percentile of the distribution, dividing the data into two equal halves. The robust standard deviation (s*) was estimated using the interquartile range (IQR) to account for variability, with $s*=\frac{1.25*\mathrm{IQR}}{1.35*\sqrt{N}}$, where *N* is the number of observations and IQR is the range between the 75th and 25th percentiles. The robust coefficient of variation (CVr %) was calculated as $\mathrm{CV}r\%=\frac{s*}{\mathrm{Me}}*100$, providing a measure of percentage variability relative to the median, useful for assessing panel reliability (Table S2).

## 3 Results and Discussion

### 3.1 Consumers Survey

A descriptive analysis of the socio‐demographic profiles for the final sample (266 consumers) is presented in Table S3. The consumers who participated in the survey were mainly women (74.8%) between 20 and 30 years old (48.9%) and 40–60 (29.7%). Most of the consumers were from the center of Italy (71.1%) and were responsible for household grocery shopping (61.1%). A first limitation of the study lies in the geographical composition of the consumer sample, with 71% of respondents residing in Central Italy. While this reflects the regional context of data collection and the strong cultural relevance of EVOO in this area, it may reduce the generalizability of the findings to the national level (Carzedda et al. 2021; De Gennaro et al. 2021). However, it is important to note that the Central Italian regions, particularly Tuscany, Umbria, Lazio, Abruzzo, and Campania, are among the most important olive oil producing and consuming areas in the country, following Puglia and Calabria. According to national agricultural data (Castellotti 2021) Central Italy hosts a high concentration of olive mills and PDO/PGI certifications, reflecting a deeply rooted EVOO culture and market relevance. Therefore, consumer awareness and engagement in these regions is especially meaningful for the aims of this study.

#### 3.1.1 Purchasing Habits

Regarding EVOO purchasing habits (Table S4), 44.9% of consumers reported consuming < 1 L per month while another 43.8% consume between 1 and 2 L per month. The most common packaging choice was a 5‐L tin (45%) followed by a 1‐L bottle (34.7%) and the price range for EVOO varies from 4 to 9 euros and above, with just 2.7% of consumers spending < 4 euros per liter. Although price remains a key factor, most consumers expressed a willingness to pay more for premium EVOO, particularly for perceived quality and health benefits (Larvoe et al. 2025).

#### 3.1.2 Label Information and Influencing Factors

**FIGURE 1:**
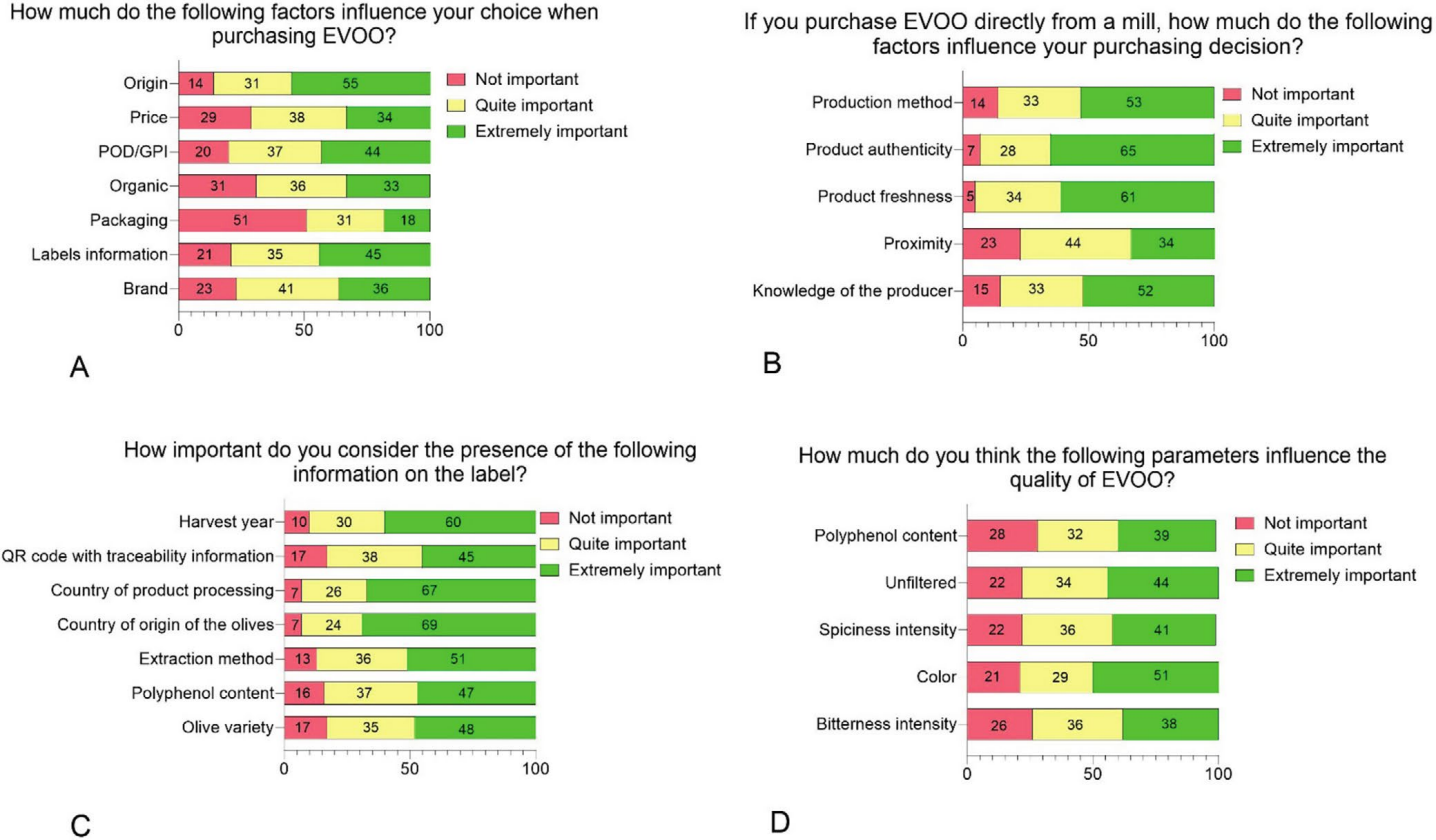
Consumer perceptions (266 consumers) of various factors influencing EVOO purchase decisions. Bars are divided into categories of importance: Not Important, Quite Important, and Extremely Important, with corresponding percentages displayed within each segment of the horizontal bar. Each panel (A, B, C, and D) corresponds to the specific questions.

A significant proportion (70.8%) of consumers reported reading label information before purchasing EVOO. The data suggest that origin certifications (PDO/PGI) and other label information strongly influence purchasing decisions (Figure 1A). By contrast, packaging was generally rated as less important, while factors such as price, organic certification, and brand held moderate influence. These preferences reflect Italy's historical and cultural connection to olive oil and the Mediterranean diet, which fosters greater consumer awareness of product quality attributes (Spognardi et al. 2021). This cultural backdrop is likely why Italian consumers are often considered among the most informed globally.

However, despite this cultural backdrop, the survey revealed notable knowledge gaps among consumers. While labels often create expectations regarding quality, many claims on labels are not fully understood by consumers. For example, health claims about polyphenol content appear to influence preferences (Mancebo‐Campos et al. 2023), yet consumers frequently lack the knowledge needed to accurately interpret such claims.

#### 3.1.3 Purchasing Context: Supermarkets vs. Direct From Producers

When consumers purchase EVOO from supermarkets or retail outlets, origin and certifications (PDO/PGI) are the most significant drivers. In contrast, when purchasing EVOO directly from oil mills, other factors influence the choice (Figure 1B). Specifically, product authenticity and product freshness lead to a higher perceived quality. Producer knowledge is another critical factor, as it probably guarantees the product quality in the consumers mind. Consumers are also influenced by the production method, which reinforces the perceived quality of the oil. Thus, while general EVOO purchases are driven by certifications, origin, and label information, direct purchases at oil mills emphasize trust, freshness, and local connections.

#### 3.1.4 Perceived Importance of Label Attributes

When asked about the importance of specific label attributes, respondents ranked several as extremely important, including harvest year, QR codes for traceability, country of origin and production, extraction method, polyphenol content, and olive variety (Figure 1C). Among these, country of origin and processing were cited most frequently (69% and 67%, respectively). This reflects the strong role of geographical origin in consumer perceptions of authenticity and quality (Caporale et al. 2006). The impact of such information is particularly relevant for traditional products, as EVOO, which communicate about typicality. Nonetheless, the results reveal a discrepancy between perceived knowledge and actual understanding. For instance, 51% of respondents believed that olive oil color was extremely important in determining quality, and 44% assigned similar importance to whether the oil was filtered or unfiltered (Figure 1D). However, this perception is misleading, as the green color is not a reliable indicator of superior quality (Arrizabalaga‐Larrañaga et al. 2021) and, unfiltered oils, though often perceived as more authentic, are more susceptible to quality deterioration due to suspended particles that can accelerate oxidation and spoilage (Cinelli et al. 2020). These results highlight a significant gap between consumers' perceived and actual knowledge of the key factors influencing EVOO quality: while many consumers believe they are well‐informed, their reliance on attributes such as color and filtration highlights misconceptions about what truly determines high‐quality oil.

#### 3.1.5 Knowledge Gaps on Technical Aspects

**FIGURE 2:**
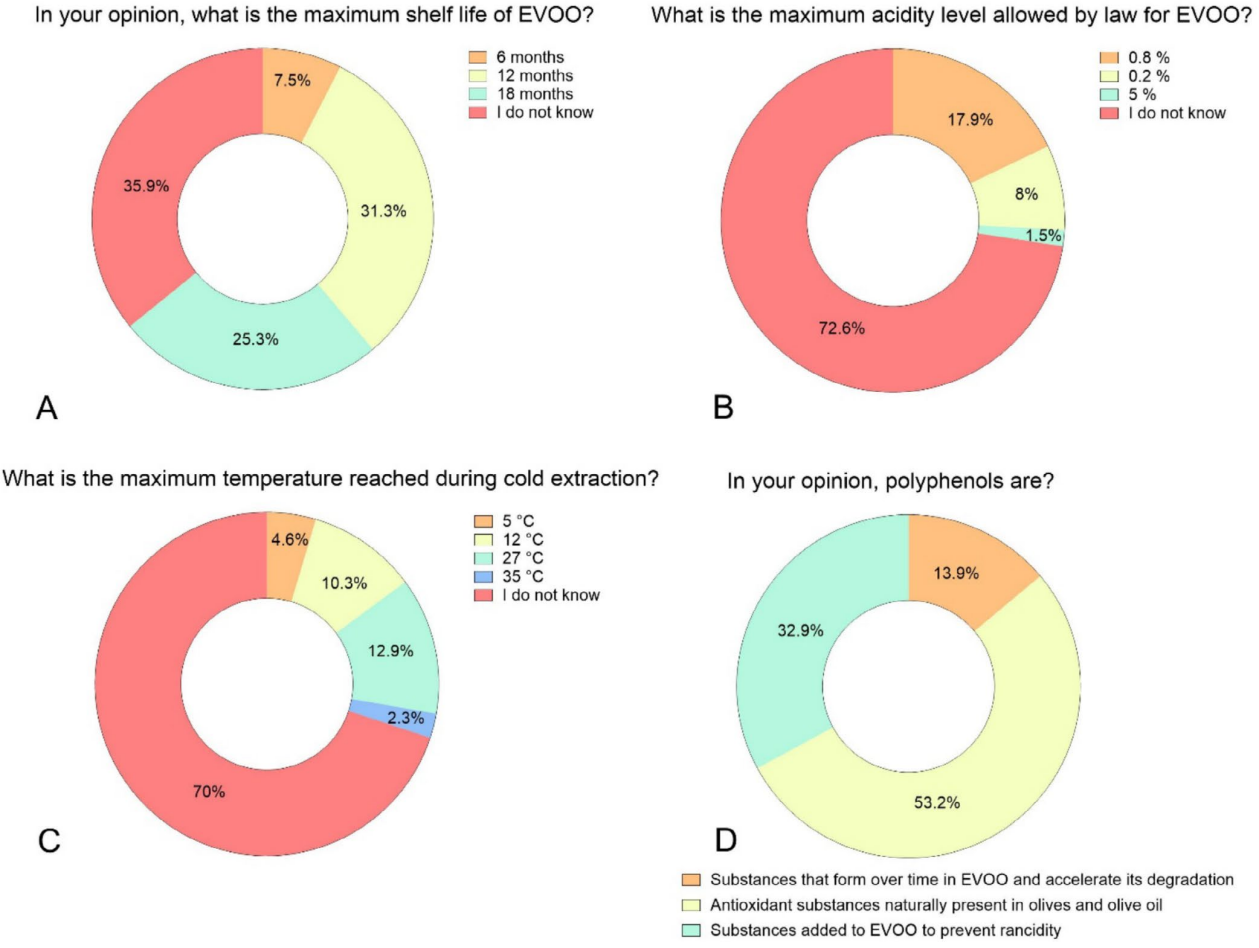
Distribution of consumer responses (% of 266 consumers) to the online survey, illustrating knowledge regarding key aspects of EVOO, including maximum shelf life (A), acidity (B), cold extraction (C), and polyphenols (D).

The knowledge gap became even more evident when consumers were asked more technical questions (Figure 2): 35.9% of the consumers were unaware about the maximum recommended shelf life for EVOO and just 31.3% correctly identified it as 12 months (Figure 2A). Moreover, 70% did not know the legal maximum acidity level allowed in EVOO classification (Figure 2B). Although 51% considered extraction method information crucial (Figure 1C), 70% admitted to having no knowledge of the actual temperature range used in cold extraction processes (Figure 2C). Moreover, while 51% correctly identified polyphenols as naturally occurring antioxidant compounds, 32.9% incorrectly believed that polyphenols are additives used to prevent rancidity (Figure 2D). As clearly evident from the consumers survey, there is a significant confusion between what consumers believe they know and value in EVOO and the factors that effectively influence its quality. This disparity can result in misalignment between perceived and actual quality, leading to unmet expectations. According to expectation‐disconfirmation theory, unmet expectations can negatively impact future perceptions of product quality (Caporale et al. 2006). If such misunderstandings are prevalent in Italy, a country with deep cultural ties to olive oil, knowledge gaps are likely to be even more pronounced in countries where olive oil consumption is less entrenched. This underlines the need for targeted consumer education to promote informed purchasing decisions and maintain trust in the EVOO market.

### 3.2 Commercial Retailed Offer

The second step focused on a commercial survey to assess the availability and completeness of label information accessible to consumers for EVOO products. The survey was conducted in five large‐scale retail outlets in Viterbo, a region of Central Italy with a solid tradition of olive cultivation. The retailers included two hypermarkets and three discount stores.

The results revealed considerable variation in both the range of EVOO products offered and the quality indicators reported on product labels, particularly between hypermarkets and discount stores. Table 2 shows the parameters assessed, including package size, price per liter, origin, harvest year, and designation of origin certification (PDO/PGI). The number of EVOO products varied widely between the retailers surveyed, with Iper B offering the most large selection (62 products) and Discount C the most limited (three products). One‐liter bottles were the most common packaging format, particularly in hypermarkets, aligning with the second most popular format identified in the consumer survey. In contrast, smaller packages (500 mL or 750 mL) were more prevalent in discount stores. While previous study reported that larger package sizes often appeal to consumers seeking better price‐per‐liter value (Boccia et al. 2024), our consumer survey indicated that packaging was considered less important in purchasing decisions.

**TABLE 2:** Parameters assessed (packaging size, price per liter, origin, harvest year, and designation of origin [DOP/IGP] certification) for the different EVOO sell in the different retailers.

Capacity	*n*	Price €/L	*n*	Origin	*n*	Year of harvest	*n*	DOP/IGP	*n*
Hypermarket A (HypA)									
0.5	4	< 7	7	IItaly	25	21/22	8	DOP	5
0.75	16	7–14	23	UE	11	22/23	4	IGP	1
1	17	> 14	9	UE and non‐UE	3	Not declared	28	None	34
> 1	3	> 20	1	Not declared	1				
Tot	40		40		40		40		40
Hypermarket B (HypB)									
0.5	11	< 7	13	Italy	45	21/22	18	DOP	9
0.75	10	7–14	33	UE	15	22/23	6	IGP	1
1	38	> 14	11	UE and non‐UE	2	Not declared	38	None	52
0.25	2	> 20	3	Not declared	0				
0.45	1	Not declared	2						
Tot	62		62		62		62		62
Discount A (DisA)									
0.5	1	< 7	3	Italy	6	21/22	4	DOP	1
0.75	5	7–14	7	UE	4	22/23	1	IGP	0
1	4	> 14	0	UE and non‐UE	0	Not declared	5	None	9
> 1	0	> 20	0	Not declared	0				
Tot	10		10		10		10		10
Discount B (DisB)									
0.5	0	< 7	3	Italy	3	21/22	1	DOP	1
0.75	3	7–14	3	UE	3	22/23	1	IGP	0
1	3	> 14	0	UE and non‐UE	0	Not declared	4	None	5
> 1	0	> 20	0	Not declared	0				
Tot	6		6		6		6		6
Discount C (DisC)									
0.5	0	< 7	3	Italy	0	21/22	0	DOP	0
0.75	3	7–14	0	UE	2	22/23	0	IGP	0
1	0	> 14	0	UE and non‐UE	1	Not declared	3	None	3
> 1	0	> 20	0	Not declared	0				
Tot	3		3		3		3		3

Price analysis revealed that EVOO products in hypermarkets were in the mid‐price range (over €7 but under €14 per liter): 57.5% of the selection at Hypermarket A and 53.2% at Hypermarket B fall within this range. Discount stores offered fewer high‐priced products, with most EVOO offerings priced below €14 per liter and none above €20. This pattern suggests a more budget‐oriented strategy in discount stores, which may prioritize affordability over premium branding. Previous studies have shown that price often acts as a heuristic for perceived quality in food products (Grunert 2005), and while high prices are sometimes associated with higher quality, price alone does not guarantee higher quality, especially in the EVOO market (Maesano et al. 2021).

A distinct trend emerged regarding the origin of the EVOO products. Oils of Italian origin predominated across all retailers: 72.6% at Iper B and 60% at Discount A. Emphasizing Italian provenance likely appeals to consumer preferences for authenticity and quality. Nevertheless, 27.5% of Iper A selection and 24.2% of Iper B selection included oils from other EU countries, reflecting the increasingly globalized nature of the EVOO market. Some discount store products did not specify a country of origin, potentially undermining perceptions of authenticity and transparency. This issue was also highlighted in our consumer survey, which found that origin labeling strongly influences purchasing decisions. These findings align with previous research demonstrating the critical role of origin labeling in consumer trust and product evaluation (Menozzi et al. 2015).

A significant number of products do not report harvest year information, with 70% of the Hyper A selection and 61.3% of the Hyper B selection not reporting a harvest year. Among those that did, most referred to the previous olive growing season, indicating that the oil was nearing the end of its recommended shelf life. Harvest year transparency is crucial, as it directly correlates with freshness, sensory, and chemical qualities (Kalua et al. 2007). Studies have shown that consumers value freshness in EVOO because older oils tend to lose health benefits and sensory appeal due to oxidation (Blekas et al. 2006). However, our survey revealed a knowledge gap: while 60% of consumers expressed appreciation for the inclusion of the harvest year on labels, 35.9% were unaware that the maximum recommended shelf life for EVOO is only 1 year. This lack of knowledge may limit the effectiveness of harvest year information in guiding informed purchasing decisions.

Finally, the presence of PDO and PGI certifications was limited. Only 12.5% of products at Hypermarket A and 14.5% at Hypermarket B carried a PDO designation, with even fewer displaying PGI certification. Among discount retailers, only one product each from Discount A and Discount B had PDO certification. This disparity in the presence of DOP/IGP could reflect differences in consumer expectations between supermarkets and discount shoppers, with the latter potentially prioritizing lower prices over quality‐related certifications (Aprile et al. 2012).

Overall, supermarkets typically offer a broader selection of EVOO products and more options with DOP/IGP certifications and Italian origin indicators, potentially attracting consumers who prioritize quality. On the other hand, discounters offer fewer options and limited information on the harvest year, which could impede informed decision‐making. This aligns with Van Doorn and Verhoef's (van Doorn and Verhoef 2011) findings, which suggest that discount shoppers are typically less informed about quality indicators, which could affect their purchase satisfaction.

### 3.3 Quality Assessment

Lastly, a quality assessment was performed on 11 EVOO samples (Section 2.1 and Table 1), selected from different retailers and price ranges, with the main objective of verifying whether oils commercially labeled as EVOO actually met the regulatory criteria required for this classification. In addition to assessing key analytical parameters (free acidity, peroxide value, polyphenol content, volatile profile, and sensory attributes), we also collected and analyzed label information, such as DOP/PGI certification, cultivar type, extraction method, and harvest year, to explore whether more informative labeling correlated with higher product quality. Lower‐priced oils (LP1–LP3) lacked all optional quality‐related details: none reported cultivar type, or extraction method beyond the legally required “extra virgin” designation, and all were EU blends without PDO/PGI certification. Medium‐priced oils showed slightly more variation: while MP1 and MP2 had limited label content, MP3 and MP4 reported additional details such as cold extraction and Italian origin, with MP4 being the only sample to declare PGI certification. Among the high‐priced oils, HP2, HP3, and HP4, purchased directly from mills, were the most transparent, all reporting cold extraction, monocultivar origin, and harvest year. HP1, a premium oil sold in a hypermarket, also report several of these declarations (i.e., extraction method, cultivar and filtration). Notably, none of the samples explicitly claimed to be “high in polyphenols,” although the presence of quality cues, such as DOP/PGI certification, cultivar origin, or extraction method, often implied superior quality.

#### 3.3.1 Free Acidity, Peroxide Value, and Total Polyphenols

Table 3 shows the free acidity, peroxide value, and total polyphenols content in the different samples. TPC ranges from 61.44 to 520.61 mg/kg, with higher values observed in most of the more expensive EVOOs (HP2, HP3, and HP4). On the other hand, HP1 showed a lower phenolic content, indicating that price alone does not always reflect high polyphenol content and antioxidant levels (Carrasco‐Pancorbo et al. 2005). Medium‐and low‐priced oils show a considerable variation in polyphenol content without a clear trend related to price range, highlighting a high variability in polyphenol levels that may result from differences in processing, cultivars, and origin of the olives rather than economic value alone. Acidity levels meet the requirements for EVOO classification (≤ 0.8% oleic acid) (European Commision 2013), ranging from 0.19% to 0.64% in all samples. The lowest acidity values (0.19% to 0.27%) were observed in high‐priced oils, suggesting a strong correlation between lower acidity and premium quality, as lower free acidity is often associated with higher freshness and reduced oxidation (Blekas et al. 2006). In contrast, medium‐ and low‐priced oils exhibit a broader range of acidity values, with several samples approaching the limit of EVOO standards. Peroxide values similarly comply with EVOO regulations (≤ 20 meq O_2_ kg^−1^) (European Commision 2013) but do not show a consistent trend with price. This observation may indicate that peroxide levels, while helpful in assessing oxidative stability during testing, do not reflect differences between economic categories (García‐González et al. 2008). Overall, all the oils labeled as EVOO meet the legal requirements imposed by EU regulation.

**TABLE 3:** Total polyphenol content (TPC, mg kg^− 1^), free fatty acids (FFA, % oleic acid), and peroxide value (PV, meq O_2_ kg^−1^) in different oil samples of different price tiers: low (LP1, 2, and 3), medium (MP1, 2, 3, and 4), and high (HP1, 2, and 3).

	TPC	FFA	PV
LP1	61.44 ± 5.22 f	0.63 ± 0.00 e	10.98 ± 0.95 b
LP2	104.49 ± 6.72 d	0.37 ± 0.00 c	12.09 ± 1.20 b
LP3	87.30 ± 4.32 e	0.40 ± 0.02 c	16.02 ± 1.32 a
MP1	127.65 ± 7.54 cd	0.38 ± 0.01 c	6.84 ± 0.97 c
MP2	94.36 ± 3.22 e	0.38 ± 0.02 c	10.64 ± 1.06 b
MP3	103.41 ± 6.22 d	0.47 ± 0.03 d	15.85 ± 1.23 a
MP4	106.20 ± 4.52 d	0.37 ± 0.01 c	7.68 ± 0.94 c
HP1	179.10 ± 4.85 c	0.27 ± 0.03 b	11.13 ± 0.98 b
HP2	520.61 ± 9.21 a	0.27 ± 0.03 b	6.99 ± 0.65 c
HP3	411.51 ± 6.32 b	0.20 ± 0.00 a	11.68 ± 1.12 b
HP4	482.03 ± 4.21 b	0.18 ± 0.00 a	10.79 ± 0.98 b

*Note:* Values are the mean of three replicates ± standard deviation. Different letters means statistically significant differences based on one‐way ANOVA and post hoc Tukey's test with *p* ≤ 0.05.

#### 3.3.2 Aromatic Composition

**FIGURE 3:**
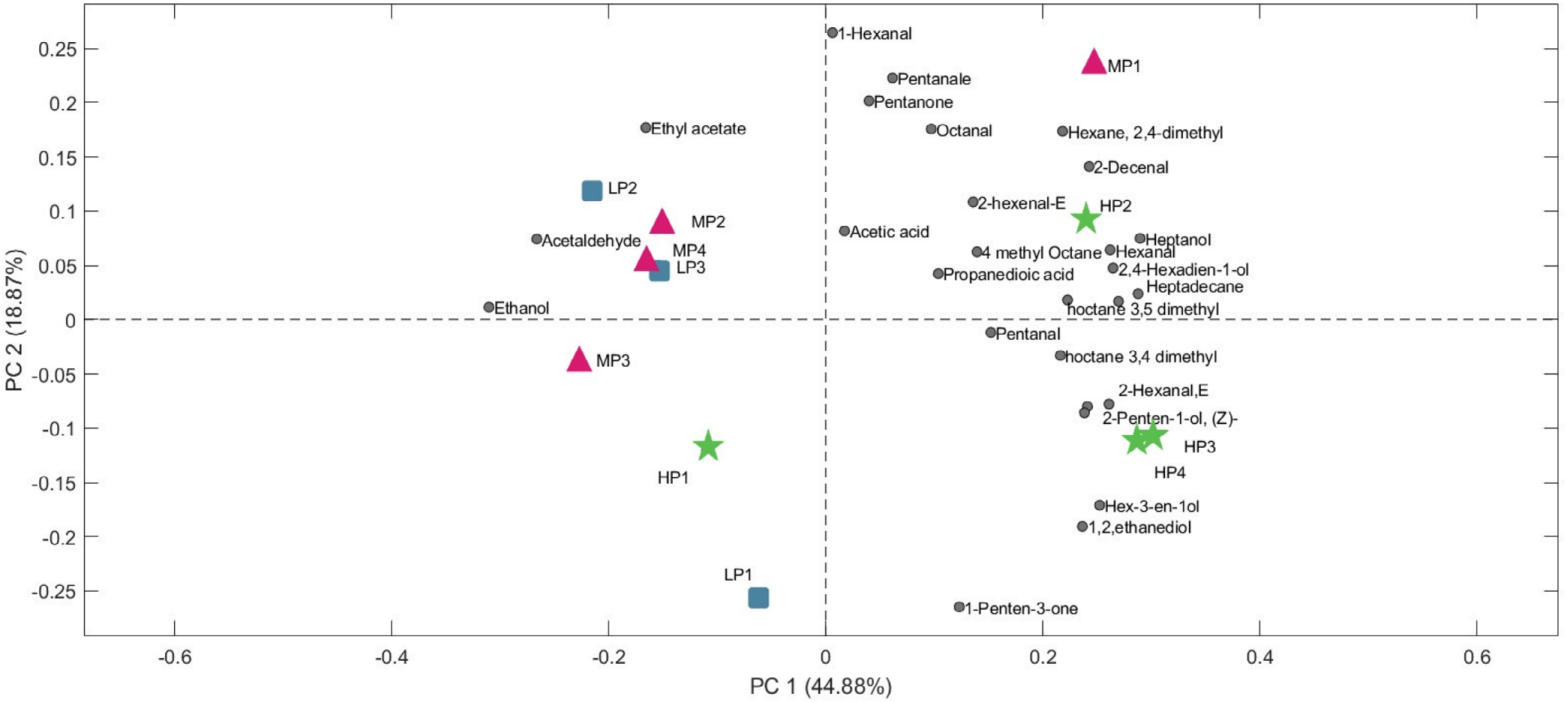
Biplot (PC1 vs. PC2) of the PCA built with autoscaled HS‐GC–MS data. Green stars: high‐priced oils (HP1–HP4); magenta triangles: medium‐priced oils (MP1–MP4); blue squares: low‐priced oils (LP1–LP3).

The aromatic profile of olive oils is another critical factor in determining EVOOs’ quality and freshness. Through HS‐GC–MS, 28 volatile compounds were identified across the EVOO samples, which were then normalized and used for a PCA, an unsupervised statistical method selected to explore natural clustering and variability within the VOC dataset. The PCA explained approximately 60% of the variance within the first two principal components (PC1: 41.88%, PC2: 18.87%). The biplot in Figure 3 showed a clear separation of oils primarily along PC2, where lower‐priced samples (LP1, 2, and 3) exhibited strong associations with ethyl acetate, acetaldehyde, acetic acid, and ethanol. These compounds, typically associated with fermentation by‐products, produce “winey‐vinegary” sensory notes. Their presence indicates that the olives likely underwent prolonged storage before processing, leading to quality deterioration (Kalua et al. 2007). Such fermentation‐related volatiles in lower‐priced oils may mask the fruity aromas typically associated with higher‐quality, premium EVOOs.

In contrast, higher‐priced samples were strongly associated with C6 aldehydes and alcohols, particularly (E)‐2‐hexenal, which is considered a marker of high‐quality EVOO. These C5 and C6 compounds, primarily derived from the lipoxygenase pathway, impart desirable herbaceous and green notes, characteristic of fresh, fruity olive oils (Aparicio and Luna 2002). The presence of these unsaturated aldehydes suggests a fresher and more complex aromatic profile.

Notably, GC–MS identifies and quantifies individual molecules but does not account for potential synergies or antagonisms among compounds, nor does it capture the effects of volatiles present below their olfactory threshold values (OTVs). These complex interactions may contribute to the richness of an oil's aroma profile and are relevant for a comprehensive understanding of sensory quality.

**FIGURE 4:**
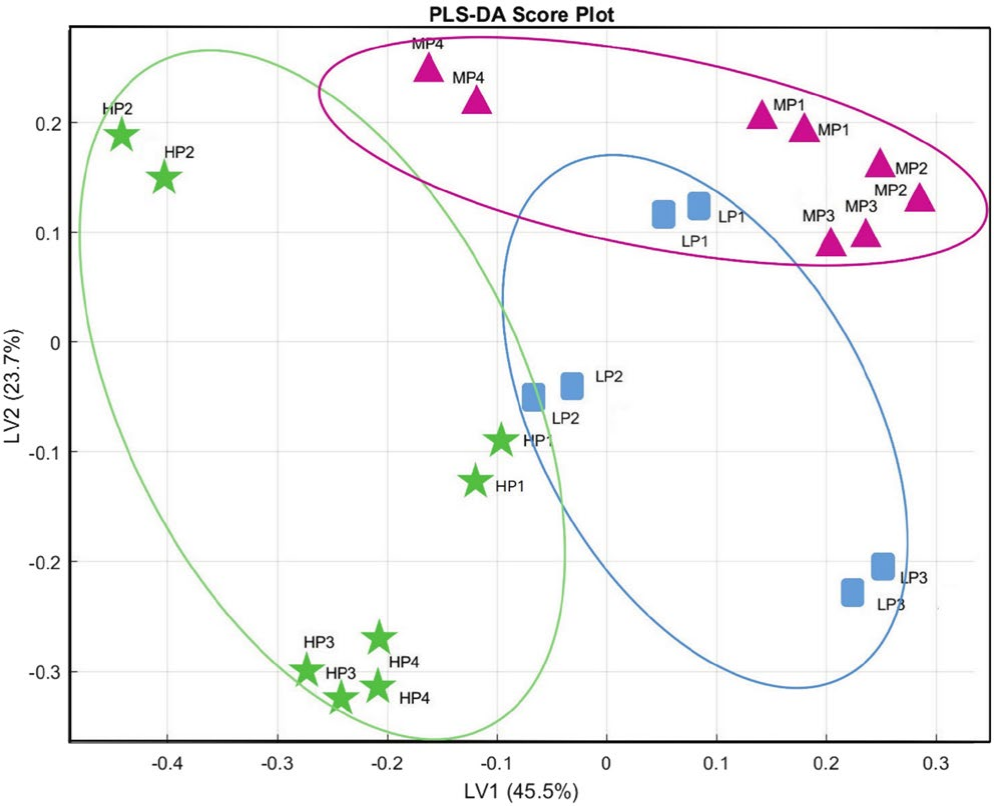
Score plot (LV1 vs. LV2) of the PLS‐DA built with E‐nose measurement autoscaled data. Green stars: high‐priced oils (HP1–HP4); magenta triangles: medium‐priced oils (MP1–MP4); blue squares: low‐priced oils (LP1–LP3).

Recent studies have confirmed the value of E‐nose systems w to improve classification accuracy, monitor oxidation, and detect fraud in EVOO (Martín‐Tornero et al. 2023). Therefore, to complement the GC–MS data, an electronic nose with 12 QMBs was employed to evaluate the global volatile fingerprint of each sample. This device, sensitive to mass changes, allowed the detection of differences in aroma profiles among samples. PLS‐DA was applied to the E‐nose data to explore the classification potential of volatile fingerprinting across price‐based olive oil categories. Unlike PCA, PLS‐DA is a supervised method that emphasizes interclass discrimination. The final model explained 69.2% of the total variance (LV1: 45.5%, LV2: 23.7%). The resulting score plot (Figure 4) reveals a partial but interpretable separation of oil samples. High‐priced oils (HP3, HP4) are distinctly clustered in the lower‐left quadrant, indicating a consistent volatile response across replicates. HP2 appears more distant along LV2, suggesting some intra‐group heterogeneity, possibly reflecting differences in specific aromatic compounds or technological processing. Interestingly, HP1 clusters closer to LP2 and LP1, aligning with its lower polyphenol content and confirming previous findings that price alone does not guarantee sensory superiority. Medium‐priced samples (MP1, MP2, MP3, MP4) appear dispersed across the central and upper‐right portions of the plot. Their scattering suggests greater aromatic variability, likely due to differences in origin, cultivar, or production scale. LP samples (LP1–LP3) are also dispersed, with LP3 shifted furthest along LV1, indicating a stronger or qualitatively different sensor response, potentially reflecting the presence of defect‐associated volatiles.

These clustering patterns are largely consistent with those observed in the GC–MS‐based PCA plot (Figure 3). In that figure, LP3, MP4, and MP2 form a dense cluster in the upper left quadrant, characterized by high loadings of ethanol, acetaldehyde, and ethyl acetate—compounds frequently associated with fermentation and sensory defects. Conversely, HP3 and HP4 occupy the lower‐right area, strongly associated with fresh green volatiles like E‐2‐hexenal, 1‐hexanol, and 2‐penten‐1‐ol, markers of higher quality. Notably, the samples most distant in Figure 4 (HP3, HP4 vs. LP3) are also maximally separated in the GC–MS PCA, reinforcing the conclusion that both analytical platforms detect coherent differences in aroma profile. This convergence between E‐nose and GC–MS confirms that the QMB sensor array captures chemically relevant variations in volatile composition. Classification accuracy (68.2%) and precision (ranging 0.625–0.75) further support the discriminative power of the E‐nose (Table S5). Most classification errors occurred between medium‐ and low‐priced samples, consistent with their overlapping chemical signatures. Overall, the combined interpretation of GC–MS and E‐nose data offers a complementary understanding of EVOO aromatic quality, with GC–MS identifying key volatiles and the E‐nose capturing holistic pattern responses (Cicia et al. 2013).

#### 3.3.3 Sensory Analysis

The classification of EVOO relies heavily on sensory analysis (Council International Olive Oil 2021, 2024). For UE regulation, EVOO must have a median of defect equal to 0 and a median of the fruity attribute higher than 0 (Council international Olive Oil 2022). The sensory analysis conducted by a trained panel on EVOOs demonstrated important discrepancies in both the intensity of positive attributes as well as the presence of defects (Table 4). High‐priced oils display intense positive attributes, including “fruity,” “pungent,” and “bitter,” although this does not always ensure the absence of defects. In particular, HP2, HP3, and HP4 rank higher for fruity and pungent intensities. On the other hand, in samples LP1, LP2, LP3, MP1, MP3, and HP1, the “rancid” defect was present. Interestingly, in these samples, volatile compounds responsible for the rancid defect (Aparicio and Luna 2002), including aldehydes and ketones, were detected. This defect, mostly associated with lipid oxidation, can occur due to prolonged exposure to air or improper storage (García‐González et al. 2008). Some samples (LP2, LP3, MP3) also exhibited “musty” or “muddy” notes. At the same time, HP1, despite being selected as a premium EVOO, showed a slight “musty” aroma attributed to methyl ketones, suggesting microbial contamination during production or storage.

**TABLE 4:** Sensory data analysis results including median (Me) and coefficient of variation (%) for evaluation of sensory profile of the different oils.

	Fruity		Bitter		Pungency		Rancid		Fusty		Winey			
	Me	CVr%	Me	CVr%	Me	CVr%	Me	CVr%	Me	CVr%	Me	CVr%	Me	CVr%
LP1	4.0	2.0	2.5	16.4	2.5	16.4	0.0	0.0	2.5	16.4	1.0	12.3	0.0	0.0
LP2	3.0	6.8	2.5	14.7	2.0	12.3	3.5	12.9	0.0	0.0	0.0	0.0	0.0	0.0
HP1	4.0	8.2	2.0	8.2	5.0	5.7	1.0	10.6	0.0	0.0	0.0	0.0	0.0	0.0
MP1	3.0	6.8	3.5	15.2	5.0	8.2	2.0	12.3	0.0	0.0	0.0	0.0	0.0	0.0
MP2	3.5	15.2	3.5	12.9	4.5	10.0	0.0	0.0	0.0	0.0	2.5	14.7	0.0	0.0
LP3	4.0	3.1	3.0	16.4	5.5	11.9	0.0	0.0	0.0	0.0	0.0	0.0	0.0	0.0
MP3	2.5	14.7	4.5	13.6	5.0	18.8	2.8	17.9	2.0	16.4	1.0	8.2	0.0	0.0
MP4	2.5	6.5	3.5	14.0	4.0	3.1	0.0	0.0	0.0	0.0	0.0	0.0	0.0	0.0
HP2	3.0	2.7	3.0	4.1	6.0	3.4	0.0	4.1	0.0	0.0	0.0	0.0	0.0	0.0
HP3	3.0	4.1	3.3	10.1	3.5	14.0	0.0	0.0	0.0	0.0	0.0	0.0	0.0	0.0
HP4	3.8	8.7	4.0	5.1	3.8	9.8	0.0	0.0	0.0	0.0	0.0	0.0	0.0	0.0

Positive sensory traits such as fruitiness, bitterness, and pungency, which are crucial characteristics of high‐quality EVOO, were found to be significantly more pronounced in the medium‐ and high‐priced samples. In contrast, low‐priced EVOOs demonstrated reduced intensity in these attributes, particularly fruitiness and pungency. These results confirm that lower‐cost oils often originate from less controlled production processes, which can affect the sensory qualities (Caporale et al. 2006). Most concerning was the detection of sensory defects, such as rancid, fusty, winey, or musty notes, that should disqualify oils from being labeled as EVOO (Council international Olive Oil 2022). These observations underscore broader structural issues affecting the EVOO retail sector. While the data show that higher‐priced EVOO samples often exhibited more favorable values for parameters such as polyphenol content, free acidity, and fruitiness, this trend was not consistent across all samples. For example, one of the most expensive oils (HP1) presented clear signs of sensory deterioration, suggesting that high price alone does not guarantee superior quality. This inconsistency points to structural weaknesses in the EVOO supply chain, particularly within LSD, where inadequate storage conditions and long shelf‐life exposure may compromise product integrity, regardless of declared quality or price tier (Torrecilla and Cancilla 2021). For example, most of the low‐priced samples (e.g., LP1 and LP2) showed fusty and winey aromas, while some medium‐priced oils (e.g., MP3) presented slight rancidity. Most of the times, these defects are due to improper storage, delayed processing, or fermentation of olives and indicate serious lapses in production and handling protocols. As previously states, according to the IOC standards, EVOO must be free of any sensory defects (Council international Olive Oil 2022). The presence of these defects in oils sold as EVOO reflects scarce quality control and a lack of enforcement of regulatory standards in the LSD. These findings highlight the limitations of relying solely on price or label information as quality indicators. Although consumers often associate higher price with higher quality, this assumption can be misleading in the absence of strict and consistently enforced regulatory standards. In particular, current EU regulations allow for the classification of oils as “extra virgin” based on a minimum set of criteria, yet our study identified several samples with sensory defects that should technically exclude them from the EVOO category. Previous studies have reported similar results, showing that a significant proportion of commercially available EVOOs fail to meet the criteria established for the extra virgin grade (Gómez‐Caravaca et al. 2016; Velasco and Dobarganes 2002). An additional observation is the variability of sensory attributes within medium‐ and high‐priced oils. While these oils generally showed better sensory profiles, the presence of minor defects or reduced positive attributes suggests that price alone is not a concrete indicator of the quality. This regulatory leniency, combined with inconsistent enforcement and limited supply chain transparency, may enable oils of substandard quality to reach the market under premium labels (Torrecilla and Cancilla 2021). Therefore, the role of price as a proxy for quality is context‐dependent and should be interpreted cautiously, especially in the retail context where preservation and compliance controls are often lacking. In light of these inconsistencies, we also examined whether label informativeness could offer additional cues to predict actual product quality (Table 1). Although the sample size limits broad generalizations, a pattern emerged in the analysis of labeling content: specific elements such as harvest year, cultivar type, and cold extraction method were frequently present in higher‐quality oils. Oils that lacked the harvest year (e.g., LP1, LP2) often exhibited sensory defects and lower polyphenol content, while those declaring cold extraction (e.g., MP3, HP2–HP4) consistently showed superior phenolic content and fewer sensory flaws. Cultivar declaration also aligned with higher sensory scores, suggesting that producers who emphasize varietal origin may be more likely to adopt quality‐oriented practices. These observations imply that not all label elements contribute equally to quality perception, certain indicators, especially harvest year and extraction method, may serve as particularly reliable cues for consumers seeking high‐quality oils. Future studies could use larger datasets to quantify these correlations more precisely and potentially advocate for their mandatory inclusion in labeling standards. However, exceptions were noted. HP1, despite declaring cold extraction and being positioned as a premium product, displayed low antioxidant levels and slight sensory defects (“musty” and “rancid”), likely due to suboptimal storage or processing conditions. This example highlights the fact that label claims do not always ensure high quality and that post‐production factors can significantly affect product integrity.

## 4 Conclusions

These observations highlight a critical issue in the EVOO market: the gap between consumer expectations and actual product quality. Consumers often rely on labels and price as proxies for quality; however, the inconsistencies identified in this study demonstrate that these indicators are not always reliable. This disconnect can undermine consumer trust and underscores the urgency of enhancing supply chain monitoring and quality assurance practices. Moreover, while the consumer survey revealed that purchasing decisions are largely influenced by certifications, geographic origin, and label information, it also exposed significant gaps in consumers' understanding of what these attributes imply for product quality. For example, although many consumers claimed to pay attention to label information such as the harvest year, production methods, and polyphenol content, few demonstrated a clear understanding of their relevance to oil quality. This misalignment between consumer expectations and actual product quality is further reinforced by the retail analysis. Many EVOO products lacked crucial label information, such as the harvest year, limiting consumers' ability to make informed purchasing decisions. Additionally, sensory analyses revealed a significant discrepancy between declared and actual quality: although all products were labeled as EVOO, many mid‐ and low‐priced oils exhibited sensory defects that are explicitly prohibited by EU regulations for EVOO classification. Notably, even higher‐priced oils displayed minor sensory defects, suggesting that a higher price does not necessarily guarantee superior quality.

While the study's limited sample size and regional focus represent potential limitations, the findings strongly suggest that incomplete transparency and gaps in consumer knowledge may contribute to the erosion of trust in the EVOO market. Given Italy's prominent role as both a leading producer and informed consumer of olive oil, these findings are particularly concerning. If such issues persist in Italy, they could signal even more significant challenges in markets with less developed consumer awareness. Addressing these gaps through stricter labeling regulations, improved quality control, and targeted consumer education will be crucial to safeguarding the integrity and reputation of the EVOO sector.

## Author Contributions

**DeSantis Diana:** conceptualization (equal), data curation (equal), methodology (equal), resources (equal), supervision (equal), writing – review and editing (equal). **Ferri Serena:** data curation (equal), formal analysis (equal), writing – review and editing (equal). **Barelli Benedetta:** data curation (supporting), formal analysis (equal), writing – review and editing (supporting). **Cagnazzi Lavinia:** data curation (supporting), formal analysis (equal), writing – review and editing (supporting). **Modesti Margherita:** conceptualization (equal), data curation (equal), formal analysis (equal), methodology (equal), writing – original draft (equal), writing – review and editing (equal).

## Ethics Statement

The research was carried out in line with the ethical standards of the Declaration of Helsinki. Participants were provided with a detailed explanation of the study's purpose, procedures, and use of the data, and they voluntarily agreed to participate via the statement “I am aware that my responses are confidential, and I agree to participate in this survey”. An affirmative reply was required to enter the survey. The study was conducted with the utmost respect for participants' rights and confidentiality. Any identifiable information collected from participants was anonymized to protect their privacy. Respondents did not receive any compensation for their participation in the study.

## Conflicts of Interest

The authors declare no conflicts of interest.

## Supporting information


**Appendix S1** Online consumers survey.

**Figure S1** Profile sheet used for EVOO sensory evaluation. COI/T.20/Doc. No15/Rev. 10 page 13.
**Table S1** HS, GC, and MS experimental conditions.
**Table S2** Median (Me), interquartile range (IQR), robust standard deviation (S*), and robust coefficient of variation (CVr%) of the sensory data analyzed using the official method coi sensory analysis of olive oil method for the organoleptic assessment of virgin olive oil, 2024.
**Table S3** Descriptive analysis of the socio‐demographic profiles of the final sample (266 consumers).
**Table S4** Descriptive analysis of the EVOO purchasing habits of the final sample (266 consumers).
**Table S5** Confusion matrix of the PLS‐DA built with E‐nose autoscaled data *N* = 2 (Figure 4). Err, total error; F1, F1‐score; FNR, false negative ratio; FPR, false positive ratio; P, precision = total positive (TP)/total positive + false positive; TNR, true negative ratio; TPR, true positive ratio.

## Data Availability

The data that support the findings of this study are available in the article or in the Appendices S1 and S2.
