# Supplementary material for: Is Retail Extra Virgin Olive Oil Truly Extra Virgin? Consumer Perceptions Versus Analytical Quality Across Price Ranges
Source: Food Sci Nutr. 2025 Jun 22;13(6):e70471. doi: 10.1002/fsn3.70471 (PMC12183118; doi:10.1002/fsn3.70471)
Supplement: Supplementary file 1 — Appendix S1 Online consumers survey. [file FSN3-13-e70471-s001.pdf]

Online consumers survey. The survey has been developed and distributed in Italian. The questionnaire has been then translated in English for scientific purposes only.

Purpose of the Questionnaire

Dear participant,

first of all, thank you for participating in this survey.

Its purpose is to conduct a consumer survey on purchasing motivations and perceptions of extra virgin olive oil (EVOO) quality. We would like you to answer the questions to the best of your ability, and remember there are no right or wrong answers. It will take between 5 and 15 minutes to complete the survey. You should be at least 18 years old to participate in this questionnaire.

Your responses are collected anonymously and according to the General Data Protection Regulation (GDPR – EU law on protection of personal data – EU 2016/679). Your responses will be treated confidentially and will be stored securely. You will not be asked to give your name and contact details. Your participation in this survey is voluntary and you can leave the survey at any point. Data of incomplete responses and/or small segments of respondent groups might not be included in the analysis. The results of the survey will be communicated through, amongst other things, scientific journals.

This questionnaire has been developed by the food technology research groups of the Department for Innovation in Biological, Agro-food, and Forestry Systems (DIBAF) at the University of Tuscia (Viterbo).

Pre-requisites

Are you aware that your responses are confidential, and do you agree to participate in this survey?

- Yes, proceed
- Not, leave the survey

Do you regularly consume extra virgin olive oil?

- Yes, proceed
- Not, leave the survey

Are you older than 18?

- Yes, proceed
- Not, leave the survey

Section 1: Consumer Profile

---

1. **Gender**

- Male

- Female
- Non-binary
- Prefer not to answer

2. Age

- 18-30
- 30-40
- 40-60
- Over 60

3. Education Level

- Primary school
- Middle school
- High school diploma
- Bachelor's degree
- Master's degree
- Doctoral degree

4. Occupation

- Student
- House keeper
- Retired
- Unemployed
- Employee
- Freelancer
- Entrepreneur
- Other (specified)

5. Conuntry of Origin

- Northern Italy
- Central Italy
- Southern Italy
- Europe
- Outside Europe

6. Municipality of Origin

- Less than 10,000 inhabitants
- Between 10,000 and 30,000 inhabitants
- Between 30,000 and 100,000 inhabitants
- More than 100,000 inhabitants

7. Family Composition

- 1 person
- 2 people
- 3 people
- 4 people
- More than 4 people

8. Are you responsible for food purchases for your family?

- Yes
- No

9. **How often do you usually shop for groceries?**

- Daily
- Weekly
- Every 15 days
- Monthly

Section 2: Purchasing Habits

---

1. **Approximately, how much EVOO does each member of your family consume per month?**

- Less than 1 liter
- 1 to 2 liters
- Over 2 to 3 liters
- Over 3 liters

2. **What is the price range per liter of the EVOO you usually purchase?**

- Less than €4
- €4 to €6
- Over €6 to €9
- Over €9

3. **What type of packaging do you usually purchase?**

- 5L tin
- 3L tin
- 2L tin
- 1L bottle
- 750mL bottle
- 500mL bottle

4. **Where do you usually purchase EVOO?**

- Supermarket
- Farm
- Oil mill
- Private producers
- Own production

5. **How many types of olive oil do you use in cooking?**

- One type for all preparations
- One for cooking and another for dressing
- More than two
- More than three

6. **Do you usually read the label information on the EVOO you purchase?**

- Yes
- No

Section 3: Purchasing Motivations Drivers and EVOO Knowledge

---

1. **How much do the following factors influence your choice when purchasing EVOO?**
   (1 = Definitely not; 6 = Definitely yes)

- Brand
- Labels
- Packaging
- Organic certification
- PDO/PGI certification
- Price
- Origin

2. **If you purchase EVOO at an oil mill, how much do the following factors influence your choice?**
   (1 = not important; 3 = extremely important)

- Producer knowledge
- Proximity
- Product freshness
- Product authenticity
- Production method

3. **How much do you think the following parameters influence the quality of EVOO?**
   (1 = not important; 3 = extremely important)

- Bitterness intensity
- Color
- Spiciness intensity
- Unfiltered
- Polyphenol content

4. **In your opinion, what is the maximum shelf life of EVOO?**

- Max 6 months
- Max 12 months
- Max 18 months
- I don’t know

5. **What type of packaging do you think is best for EVOO preservation?**

- Clear glass
- Dark glass
- Aluminum tin
- Plastic bottle

6. **In your opinion, do the terms "first cold pressing" and "cold extraction" mean the same thing?**

- Yes
- No

7. **What is the maximum temperature reached during cold extraction?**

- 5°C
- 15°C
- 27°C
- 35°C
- I don’t know

8. What is the maximum acidity level allowed by law for EVOO?

- 0.8%
- 2%
- 5%
- I don't know

9. In your opinion, polyphenols are:

- Substances that form over time in EVOO and accelerate its degradation
- Antioxidant substances naturally present in olives and olive oil
- Substances added to EVOO to prevent rancidity

10. How important do you consider the presence of the following information on the label?
(1 = not important; 3 = extremely important)

- Olive variety
- Polyphenol content
- Extraction method
- Country of origin of the olives
- Country of product processing
- QR code with traceability information
- Harvest year
