# Supplementary material for: Is Retail Extra Virgin Olive Oil Truly Extra Virgin? Consumer Perceptions Versus Analytical Quality Across Price Ranges
Source: Food Sci Nutr. 2025 Jun 22;13(6):e70471. doi: 10.1002/fsn3.70471 (PMC12183118; doi:10.1002/fsn3.70471)
Supplement: Supplementary file 2 — Figure S1 Profile sheet used for EVOO sensory evaluation. COI/T.20/Doc. No15/Rev. 10 page 13. Table S1 HS, GC, and MS experimental conditions. Table S2 Median (Me), interquartile range (IQR), robust standard deviation (S*), and robust coefficient of variation (CVr%) of the sensory data analyzed using the official method coi sensory analysis of olive oil method for the organoleptic assessment of virgin olive oil, 2024. Table S3 Descriptive analysis of the socio‐demographic profiles of the final sample (266 consumers). Table S4 Descriptive analysis of the EVOO purchasing habits of the final sample (266 consumers). Table S5 Confusion matrix of the PLS‐DA built with E‐nose autoscaled data N = 2 (Figure 4). Err, total error; F1, F1‐score; FNR, false negative ratio; FPR, false positive ratio; P, precision = total positive (TP)/total positive + false positive; TNR, true negative ratio; TPR, true positive ratio. [file FSN3-13-e70471-s002.docx]

Table S1 HS, GC and MS experimental conditions.

| **HS** | **Turbomatrix HS-40** |
| --- | --- |
| Headspace pressure | 20 psi |
| Thermostat vial temperature | 60 °C |
| Thermostat time | 15 min |
| Needle temperature | 70 °C |
| Transfer line temperature | 80 °C |
| Vial pressurize time | 1 min |
| Injection time | 0.02 min |
| **GC** | **PerkinElmer Clarus 680 GC** |
| Column | Elite capillary column (30m x 0,25mm x 0.25µm) |
| Injection temperature | 220 °C |
| Injection type | splitless |
| Carrier gas | Nitrogen, constant pressure (25 psi) |
| Oven programme | 40 °C for 2 min, 5 °C/min to 110 °C hold for 3 min, 10 °C/min to 230 °C, hold for 2 min |
| Run time | 33 min |
| Withdraw time | 0.20 min |
| **MS** | **PerkinElmer Clarus SQ 8 GC/MS** |
| Ionisation mode | EI |
| GC inlet line temperature | 200 °C |
| Ion source temperature | 180 °C |
| Function type | Full scan |
| Scan range | m/z 35:400 |
| Scan time | 0.20 s |
| Interscan delay time | 0.05 s |


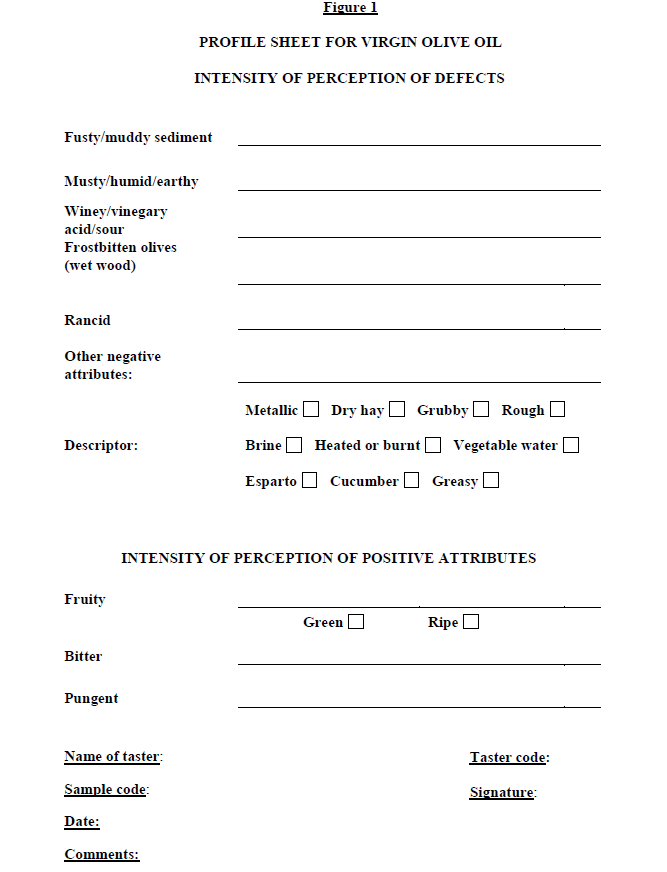


Figure S1 Profile sheet used for EVOO sensory evaluation. COI/T.20/Doc. No15/Rev.2018- 10 page 13

Table S2 Median (Me), interquartile range (IQR), robust standard deviation (S∗) and robust coefficient of variation (CVr%) of the sensory data analyzed using the official method coi sensory analysis of olive oil method for the organoleptic assessment of virgin olive oil, 2024.

|  |  | **LP1** | **LP2** | **LP3** | **MP1** | **MP2** | **MP3** | **MP4** | **HP1** | **HP2** | **HP3** | **HP4** |
| --- | --- | --- | --- | --- | --- | --- | --- | --- | --- | --- | --- | --- |
| **Fruity** | **Me** | 4.00 | 3.00 | 4.00 | 3.00 | 3.50 | 2.50 | 2.50 | 4.00 | 3.00 | 3.00 | 3.75 |
|  | **IQR** | 0.25 | 0.63 | 0.38 | 0.63 | 1.63 | 1.13 | 0.50 | 1.00 | 0.25 | 0.38 | 1.00 |
|  | **S*** | 0.08 | 0.20 | 0.12 | 0.20 | 0.53 | 0.37 | 0.16 | 0.33 | 0.08 | 0.12 | 0.33 |
|  | **CVr%** | 2.05 | 6.82 | 3.07 | 6.82 | 15.20 | 14.73 | 6.55 | 8.18 | 2.73 | 4.09 | 8.73 |
| **Bitter** | **Me** | 2.50 | 2.50 | 3.00 | 3.50 | 3.50 | 4.50 | 3.50 | 2.00 | 3.00 | 3.25 | 4.00 |
|  | **IQR** | 1.25 | 1.13 | 1.50 | 1.63 | 1.38 | 1.88 | 1.50 | 0.50 | 0.38 | 1.00 | 0.63 |
|  | **S*** | 0.41 | 0.37 | 0.49 | 0.53 | 0.45 | 0.61 | 0.49 | 0.16 | 0.12 | 0.33 | 0.20 |
|  | **CVr%** | 16.37 | 14.73 | 16.37 | 15.20 | 12.86 | 13.64 | 14.03 | 8.18 | 4.09 | 10.07 | 5.12 |
| **Pungency** | **Me** | 2.50 | 2.00 | 5.50 | 5.00 | 4.50 | 5.00 | 4.00 | 5.00 | 6.00 | 3.50 | 3.75 |
|  | **IQR** | 1.25 | 0.75 | 2.00 | 1.25 | 1.38 | 2.88 | 0.38 | 0.88 | 0.63 | 1.50 | 1.13 |
|  | **S*** | 0.41 | 0.25 | 0.65 | 0.41 | 0.45 | 0.94 | 0.12 | 0.29 | 0.20 | 0.49 | 0.37 |
|  | **CVr%** | 16.37 | 12.28 | 11.90 | 8.18 | 10.00 | 18.82 | 3.07 | 5.73 | 3.41 | 14.03 | 9.82 |
| **Rancid** | **Me** | 0.00 | 3.50 | 0.00 | 2.00 | 0.00 | 2.75 | 0.00 | 1.00 | 1.00 | 0.00 | 0.00 |
|  | **IQR** | 0.00 | 1.38 | 0.00 | 0.75 | 0.00 | 1.50 | 0.00 | 0.33 | 0.13 | 0.00 | 0.00 |
|  | **S*** | 0.00 | 0.45 | 0.00 | 0.25 | 0.00 | 0.49 | 0.00 | 0.11 | 0.04 | 0.00 | 0.00 |
|  | **CVr%** | 0.00 | 12.86 | 0.00 | 12.28 | 0.00 | 17.86 | 0.00 | 10.64 | 4.09 | 0.00 | 0.00 |
| **Fusty-muddy** | **Me** | 2.50 | 0.00 | 0.00 | 0.00 | 0.00 | 2.00 | 0.00 | 0.00 | 0.00 | 0.00 | 0.00 |
|  | **IQR** | 1.25 | 0.00 | 0.00 | 0.00 | 0.00 | 1.00 | 0.00 | 0.00 | 0.00 | 0.00 | 0.00 |
|  | **S*** | 0.41 | 0.00 | 0.00 | 0.00 | 0.00 | 0.33 | 0.00 | 0.00 | 0.00 | 0.00 | 0.00 |
|  | **CVr%** | 16.37 | 0.00 | 0.00 | 0.00 | 0.00 | 16.37 | 0.00 | 0.00 | 0.00 | 0.00 | 0.00 |
| **Winey-vinegary** | **Me** | 1.00 | 0.00 | 0.00 | 0.00 | 2.50 | 1.00 | 0.00 | 0.00 | 0.00 | 0.00 | 0.00 |
|  | **IQR** | 0.38 | 0.00 | 0.00 | 0.00 | 1.13 | 0.25 | 0.00 | 0.00 | 0.00 | 0.00 | 0.00 |
|  | **S*** | 0.12 | 0.00 | 0.00 | 0.00 | 0.37 | 0.08 | 0.00 | 0.00 | 0.00 | 0.00 | 0.00 |
|  | **CVr%** | 12.28 | 0.00 | 0.00 | 0.00 | 14.73 | 8.18 | 0.00 | 0.00 | 0.00 | 0.00 | 0.00 |
| **Musty-Earthy** | **Me** | 0.00 | 0.00 | 0.00 | 0.00 | 0.00 | 0.00 | 0.00 | 0.00 | 0.00 | 0.0 | 0.00 |
|  | **IQR** | 0.00 | 0.00 | 0.00 | 0.00 | 0.00 | 0.00 | 0.00 | 0.00 | 0.00 | 0.0 | 0.00 |
|  | **S*** | 0.00 | 0.00 | 0.00 | 0.00 | 0.00 | 0.00 | 0.00 | 0.00 | 0.00 | 0.00 | 0.00 |
|  | **CVr%** | 0.00 | 0.00 | 0.00 | 0.00 | 0.00 | 0.00 | 0.00 | 0.00 | 0.00 | 0.00 | 0.00 |

Table S3 Descriptive analysis of the socio-demographic profiles of the final sample (266 consumers)

| ***Socio-demographic characteristics*** | Percentage (%) |
| --- | --- |
| **Gender** | |
| Female | 74.8 |
| Male | 25.2 |
| Non binary | 0 |
| I prefer not to answer | 0 |
| **Age** | |
| 18-30 | 48.9 |
| 30-40 | 13.2 |
| 40-60 | 29.7 |
| Over 60 | 8.3 |
| **Education level** | |
| Primary school | 0 |
| Middle school | 0 |
| High school diploma | 44.7 |
| Bachelor's degree | 24.8 |
| Master's degree | 25.6 |
| Doctoral degree | 4.9 |
| **Occupation** | |
| Student | 26.7 |
| House keeper | 4.1 |
| Retired | 4.5 |
| Unemployed | 3 |
| Employee | 42 |
| Freelancer | 11.3 |
| Entrepreneur | 38 |
| Other | 8.4 |
| **Country of origin** | |
| Northern Italy | 12.4 |
| Central Italy | 71.1 |
| Southern Italy | 15 |
| Europe | 1.1 |
| Outside Europe | 0.4 |
| **Municipality of Origin** | |
| Less than 10,000 inhabitants | 25.3 |
| Between 10,000 and 30,000 inhabitants | 20.4 |
| Between 30,000 and 100,000 inhabitants | 35.8 |
| More than 100,000 inhabitants | 18.6 |
| **Family composition** | |
| 1 person | 9.1 |
| 2 people | 23.1 |
| 3 people | 22.7 |
| 4 people | 36 |
| More than 4 people | 10.6 |
| **Are you responsible for food purchases for your family** | |
| Yes | 61.1 |
| Not | 38.9 |
| **How often do you usually shop for groceries?** | |
| Daily | 23.9 |
| Weekly | 64 |
| Every 15 days | 10.2 |
| Monthly | 1.9 |

Table S4 Descriptive analysis of the EVOO purchasing habits of the final sample (266 consumers)

| ***EVOO Purchasing Habits*** | Percentage (%) |
| --- | --- |
| **Quantity of EVOO consumed per month** | |
| Less than 1 liter | 44.9 |
| 1 to 2 liters | 43.8 |
| Over 2 to 3 liters | 6.4 |
| Over 3 liters | 4.9 |
| **Price range per liter tipically paid for EVOO** | |
| Less than €4 | 2.7 |
| €4 to €6 | 22.1 |
| Over €6 to €9 | 39.9 |
| Over €9 | 35.3 |
| **Type of packaging tipically purchased** | |
| 5L tin | 45.9 |
| 3L tin | 6.9 |
| 2L tin | 4.6 |
| 1L bottle | 34.7 |
| 750mL bottle | 6.9 |
| 500mL bottle | 0.8 |
| **Usual place of purchase for EVOO** | |
| Supermarket | 29.2 |
| Farm | 12.5 |
| Oil mill | 8.7 |
| Private producers | 24.6 |
| Own production | 25 |
| **Number of types of olive oil used in cooking** | |
| One type for all preparations | 49.2 |
| One for cooking and another for dressing | 37.2 |
| More than two | 12 |
| More than three | 1.5 |
| **Reading of label information on purchased EVOO** | |
| Yes | 70.8 |
| No | 29.2 |

Table S5 Confusion matrix of the PLS-DA built with e-nose autoscaled data N=2 (figure 4). Err, total error; FNR, false negative ratio; FPR, false positive ratio; P, precision = total positive (TP)/total positive + false positive; TNR, true negative ratio; TPR, true positive ratio.

| \| Class \| TPR \| FPR \| TNR \| FNR \| ERR \| Precision \| \| --- \| --- \| --- \| --- \| --- \| --- \| --- \| \| HP \| 0.75 \| 0.28 \| 0.71 \| 0.25 \| 0.22 \| 0.75 \| \| LP \| 0.62 \| 0.28 \| 0.71 \| 0.37 \| 0.31 \| 0.62 \| \| MP \| 0.66 \| 0.28 \| 0.71 \| 0.33 \| 0.22 \| 0.66 \|   Confusion matrix | | |  |
| --- | --- | --- | --- | --- | --- | --- | --- | --- | --- | --- | --- | --- | --- | --- | --- | --- | --- | --- | --- | --- | --- | --- | --- | --- | --- | --- | --- | --- | --- | --- | --- |
|  | Pred_HP | Pred_LP | Pred_MP |
| **True_HP** | 6 | 0 | 2 |
| **True_LP** | 2 | 5 | 1 |
| **True_MP** | 0 | 2 | 4 |
